# Towards Next Generation Biomarkers in Natural Killer/T-Cell Lymphoma

**DOI:** 10.3390/life11080838

**Published:** 2021-08-16

**Authors:** Jason Yongsheng Chan, Jing Quan Lim, Choon Kiat Ong

**Affiliations:** 1 Division of Medical Oncology, National Cancer Centre Singapore, Singapore 169610, Singapore; 2 SingHealth Duke-NUS Blood Cancer Centre, Singapore 169857, Singapore; 3 Lymphoma Genomic Translational Research Laboratory, Division of Cellular and Molecular Research, National Cancer Centre Singapore, Singapore 169610, Singapore; lim.jing.quan@nccs.com.sg; 4 Cancer and Stem Cell Biology, Duke-NUS Medical School, Singapore 169857, Singapore; 5 Genome Institute of Singapore, A*STAR (Agency for Science, Technology and Research), Singapore 138672, Singapore

**Keywords:** precision oncology, PD-L1, immunotherapy, prognosis, EBV

## Abstract

Natural killer/T-cell lymphoma (NKTCL) is an Epstein–Barr virus-associated non-Hodgkin lymphoma linked to an aggressive clinical course and poor prognosis. Despite an improvement in survival outcomes with the incorporation of novel agents including immune checkpoint inhibitors in the treatment of NKTCL, a significant proportion of patients still relapse or remain refractory to treatment. Several clinical prognostic models have been developed for NKTCL patients treated in the modern era, though the optimal approach to risk stratification remains to be determined. Novel molecular biomarkers derived from multi-omic profiling have recently been developed, with the potential to improve diagnosis, prognostication and treatment of this disease. Notably, a number of potential biomarkers have emerged from a better understanding of the tumor immune microenvironment and inflammatory responses. This includes a recently described 3′UTR structural variant in the *PD-L1* gene, which confers susceptibility to checkpoint immunotherapy. In this review, we summarize the biomarker landscape of NKTCL and highlight emerging biomarkers with the potential for clinical implementation.

## 1. Introduction

Natural killer/T-cell lymphoma (NKTCL) is an aggressive subtype of non-Hodgkin lymphoma with a propensity for extranodal involvement. Generally considered to be a rare cancer in the West, NKTCL demonstrates a geographic distribution that is predominant in East Asia and South America [1]. Clinically, NKTCL is most frequently characterized by involvement of the nasal cavity, and the neoplastic lymphoid cells are invariably infected with Epstein–Barr virus (EBV). In the past decade, the standard treatment for NKTCL has made steady progress with the adoption of L-asparaginase-based chemotherapy regimens, including SMILE (dexamethasone, methotrexate, ifosfamide, L-asparaginase, and etoposide) [2,3], P-GEMOX (pegaspargase, gemcitabine, and oxaliplatin) [4], DDGP (dexamethasone, cisplatin, gemcitabine, and pegaspargase) [5], and AspaMetDex (pegaspargase, methotrexate, and dexamethasone) [6]. More recently, newer therapies, such as immune checkpoint inhibitors and histone deacetylase (HDAC) inhibitors, have emerged in the armamentarium against NKTCL [7]. Despite improvements in survival outcomes with the incorporation of these agents in the management of NKTCL, a significant proportion of patients still relapse or remain refractory to treatment. Furthermore, these multi-agent/multimodality regimens often carry significant toxicities including severe myelosuppression and hypersensitivity reactions. An optimized approach to prognostication and risk stratification is thus warranted so as to provide the best outcomes while minimizing adverse effects. In this review, we summarize the various model systems developed for this purpose and highlight novel biomarkers with the potential for clinical implementation (Table 1).

## 2. Clinical Risk Stratification and Prognostication Models for NK/T-Cell Lymphoma

Contemporary treatment of localized NKTCL with radiotherapy and chemotherapy results in a five-year overall survival rate of over 70% [17], while patients with advanced disease fare significantly worse with survival rates under 50% [7]. Several clinical features have been recognized to confer adverse prognosis, including extranasal compared to nasal subtypes [18], as for primary sites involving the gastrointestinal tract [19] and skin [20]. Recently, several prognostic scoring systems have been developed for NKTCL patients treated in the modern era, moving on from more traditional models including the International Prognostic Index (IPI) and Korean Prognostic Index (KPI) [21,22,23]. One of the first of such systems to be proposed was the Prognostic Index of Natural Killer lymphoma (PINK) model, which was derived from a retrospective study of patients treated with non-anthracycline-based regimens in the International NK/T-Cell Lymphoma Project. A total of four risk factors: age more than 60 years, stage III/IV disease, distant lymph node involvement and non-nasal subtype were strongly correlated with worse survival. The addition of detectable pre-treatment EBV DNA levels to the PINK model (PINK-E) further separated patients into distinctive prognostic groups [24]. More recently, a new central nervous system-prognostic index of natural killer (CNS-PINK) model was developed for the prediction of CNS relapse in extranodal NKTCL, by incorporating two or more extranodal sites involvement into the PINK model [25]. In a multicenter retrospective study, a high peripheral blood neutrophil–lymphocyte ratio (NLR) was shown to confer poor survival, in addition to age more than 60 years, stage III/IV disease, as well as the presence of B symptoms (NABS model) [26]. Similarly, another prognostic model, the Chinese Prognostic Index for Natural Killer Cell Lymphoma (C-PINK), which consisted of three risk factors (disease stage, hemoglobin concentration below 100 g/L, and local invasiveness) was also described [27].

On top of these clinical factors, a recent meta-analysis demonstrated that several indices derived from fluorodeoxyglucose-positron emission tomography/computerized tomography (FDG-PET/CT) imaging could predict survival outcomes in patients with extranodal NKTCL, suggesting that FDG-PET/CT is not only useful for the assessment of treatment response but also potentially valuable for the prognostication in clinical practice [28]. The inclusion of imaging-based indicators such as SUVmax values and Deauville scores into clinical prognostic models have been suggested to complement clinical prognostic models [29]. Interestingly, PET/CT imaging patterns may also predict tumor immune microenvironment subtype in NKTCL, with the immune-silenced PET/CT pattern conferring worse overall survival compared to others [30].

**Figure 1:**
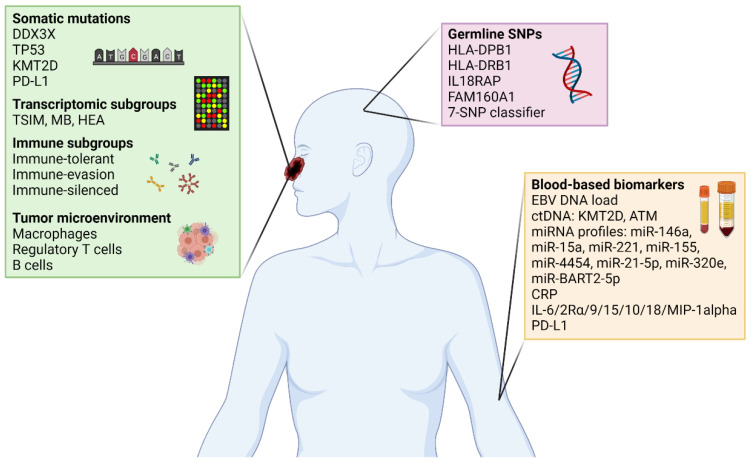
Emerging biomarker landscape for NKTCL.

While these prognostic models have improved over the years and may provide risk stratification for patients with NKTCL to a certain degree, there is probably still some way to go before they can achieve the level of consistency to enable risk-adapted approaches for treatment. Several other disease or host-related factors, including inflammatory responses, hematological indices [31,32,33,34], as well as nutritional indices [35,36,37,38,39,40], have been shown to affect outcomes in patients with NKTCL as well, and could possibly be integrated into the prognostic models. Nonetheless, these clinical prognostic systems, while inexpensive and easily accessible, ultimately suffer from modest discriminatory ability [23]. With advances in the understanding of the molecular pathobiology of NKTCL, incorporating novel molecular biomarkers may represent a potential strategy to further refine these prognostic models in order for their adoption in clinical decision making (Figure 1).

## 3. Immunohistochemical Protein Expression as Potential Biomarkers

Apart from clinical features, several biomarkers have been proposed based on protein expression on tumor cells or features in the microenvironment using immunohistochemical assays. However, the search for reliable and consistent biomarkers has been challenging due to the limitations of study design, with most being retrospective studies consisting of small patient cohorts receiving heterogeneous treatments. Differences in assigning cut-off values defining positive expression, as well as inter-observer variation, further add to difficulties in interpreting the results. For example, tumor expression of CD30, a member of the tumor necrosis factor (TNF) receptor family, has been investigated in several studies. While some studies have correlated CD30 positivity with inferior survival outcomes [41,42], others have instead demonstrated favorable results [43,44,45] or no association [46,47]. CD38, a transmembrane glycoprotein strongly expressed in NKTCL, has been associated with poor prognosis in one study [48]. The prognostic utility of these biomarkers in NKTCL remains unclear and will need to be further validated.

Ki-67 is a nuclear and nucleolar protein expressed in proliferating cells but not resting cells, and high expression levels have been demonstrated to be associated with extra-aerodigestive tract primary site, B symptoms (any of the following: unexplained fever >38 °C, drenching night sweats, or loss of >10% body weight within 6 months), and tumor bulk [49], conferring worse survival outcomes [49,50]. In keeping with these findings, expression profiles of cell cycle related proteins in NKTCL, specifically p-ATM (phospho-ATM Serine/Threonine Kinase) and CHK2 (Checkpoint kinase 2), were recently shown to correlate with worse overall survival [51]. Chen et al. showed that human trophoblastic cell surface antigen 2 (Trop2), a tumor-related protein with oncogenic functions, was overexpressed in NKTCL and correlated with adverse survival outcomes [52]. More recently, overexpression of enhancer of zeste homolog 2 (EZH2), an H3K27-specific histone methyltransferase with an oncogenic role in NKTCL, was shown to be significantly associated with higher tumor cell proliferation, advanced stage, and higher risk of death [53]. In a study on NKTCL and PTCL, expression of phosphatidylinositol 3-kinase (PIK3) isoforms was evaluated and, in particular, high PIK3α expression was significantly associated with poor survival [54].

The EBV-encoded proteins, latent membrane proteins (LMP), LMP1, and LMP2A have been examined as potential prognostic biomarkers in NKTCL. Results from several studies, however, have been conflicting. High expression of LMP1 has been associated with worse survival in two studies [55,56] but, conversely, correlated with better outcomes in two other studies [57,58]. LMP2A has been associated with poorer survival [56]. Activation of the NF-κB pathway, which is downstream of LMP1 signaling, has been correlated with chemoresistance and worse survival in NKTCL [59]. High expression of Y box binding protein 1 (YB-1), regulated by LMP1 and NF-κB, has similarly been shown to increase the potential for relapse, and conferred poor disease-free survival and reduced overall survival [60].

Taken together, results from studies evaluating histopathological protein expression in NKTCL and their prognostic significance, while promising, should be regarded as hypothesis generating and their clinical utility will require further validation.

## 4. Immune Microenvironment and Inflammatory Responses

The tumor immune microenvironment of NKTCL has been suggested to influence disease biology and clinical outcomes. A high number of tumor-associated macrophages have been shown to be associated with a higher Ki-67 proliferative index [61]. In keeping with this observation, a high degree of tumor-associated macrophages at diagnosis correlated with worse clinicopathological features, such as B symptoms and elevated serum LDH (lactate dehydrogenase) levels, as well as lower complete remission rates and dismal survival [62,63]. On the other hand, high levels of tumor-infiltrating FOXP3-positive regulatory T-cells showed prolonged overall and progression-free survival [64]. Patients with a high density of CD20+ B lymphocytes had early stage cancer and the tumors contained a low Ki-67 index. High infiltration of B lymphocytes in the tumor tissues correlated with better overall survival [65].

Apart from tumor-infiltrating immune cells, various pro-inflammatory cytokines and interleukins (IL) expressed within the tumor microenvironment or circulation may also affect patient outcomes. C-reactive protein (CRP) is an acute-phase protein secreted by hepatocytes during the inflammatory response and is regulated by pro-inflammatory cytokines. NKTCL patients with elevated serum CRP levels have been associated with adverse clinical characteristics and worse survival outcomes [18,66]. Likewise, other inflammatory factors in the serum such as ferritin [67], ORM1 (Orosomucoid-1), S100A9 [68], 14-3-3 epsilon [69] have been shown to be associated with poor survival. In recent studies, high IL-6 expression levels, both in the tumor microenvironment [70] and serum [71] were correlated with poor prognosis. Other cytokines associated with worse prognosis include high serum IL-2Rα [72], IL-9 [73], IL-15 [74], IL-10 [75], macrophage inflammatory protein 1 alpha (MIP-1alpha) [76], and IL-18 [77]. The mechanisms underlying the relationship between these pro-inflammatory cytokines and prognosis in NKTCL remain unclear. These inflammatory cytokines may potentially activate pro-oncogenic pathways, enhance tumor cell proliferation, and/or confer resistance to drug-evoked apoptosis [18,70]. IL-6 for example, has been suggested to activate the JAK/STAT pathway and also upregulate the expression of programmed cell death ligand-1 (PD-L1), thereby conferring immunosuppression in the microenvironment of NKTCL [70]. Future studies are needed to confirm the exact pathobiological mechanisms.

Targeting the Programmed cell death 1 (PD-1)/PD-1 ligand 1 (PD-L1) immune checkpoint pathway has emerged as a promising strategy for NKTCL treatment, and several studies have been conducted to evaluate its prognostic significance as well. In one study, PD-L1 expression on NKTCL tumor cells was shown to be an independent, favorable prognostic marker for overall survival in advanced NKTCL [78]. However, several others have shown either the opposite [79,80,81,82] or no effect [83]. These varying results may be due to a lack of standard guidelines for the interpretation of PD-L1 expression in NKTCL, as well as the use of different anti-PD-L1 antibodies and variable threshold cut-offs for positive staining [84]. Most importantly, the interpretation of its prognostic significance has to be considered in the context of the treatment type received by the patient cohort. In parallel, circulating PD-L1 analytes have been investigated in several studies [79,80,85]. In early stage NKTCL patients treated with asparaginase, patients with high pre-treatment serum soluble PD-L1 (sPD-L1) levels were associated with poor treatment response and high post-treatment levels and had shorter progression-free survival and overall survival [85]. In another study on early stage NKTCL patients treated with induction chemotherapy followed by consolidative radiotherapy, patients who had a high concentration of serum sPD-L1 showed lower complete response rates to treatment and poorer survival than those with a low sPD-L1 concentration [79]. Levels of PD-L1 mRNA in peripheral blood mononuclear cells and sPD-L1 were significantly associated with several adverse clinicopathological factors [86]. High pre-treatment plasma exosomal PD-L1 concentration was associated with higher SUVmax level and recurrence rate. Similarly, high sPD-L1 group was also associated with some adverse clinical parameters, including advanced stage, elevated LDH levels, B symptoms, high IPI score, and PINK score [87]. It has been suggested that soluble forms of ligands are often generated by the proteolytic cleavage of membrane-bound proteins, including PD-L1. sPD-L1 levels have been positively correlated with the total number of PD-L1-expressing tumor cells and may thus be reflective of tumor load. In addition, sPD-L1 may also be produced by immune cells, potentially signifying a suppressed T-cell mediated immune response in the tumor microenvironment [85,86,87].

## 5. Molecular Biomarkers for Diagnosis and Prognostication

Our understanding of the molecular landscape of NKTCL has greatly improved over the past years through advances in multi-omic profiling technologies as well as multinational collaboration, and efforts continue to be placed on their potential translational utility as biomarkers to facilitate the precise diagnosis, prognosis, and treatment of patients with NKTCL. Many of the early molecular studies on NKTCL were focused on the role of EBV in its pathogenesis and clinical behavior [88,89,90,91]. Presently, NKTCL tumor cells are thought to be invariably infected by EBV and contemporary diagnosis has to be supported by the presence of EBV, typically by in-situ hybridization [92]. As such, circulating EBV DNA in the blood or plasma from apoptotic tumor cells is an intuitive consequence of tumor load [93] and several studies have established EBV DNA in the blood as a significant independent predictor of poor prognosis [94,95,96,97,98,99].

Early studies in the Japanese population showed that HLA-A*02:01 was associated with a reduced risk of NKTCL [100]. More recently, genome-wide association studies revealed that germline single-nucleotide polymorphisms (SNPs) at the loci of the *HLA-DPB1*, *HLA-DRB1*, and *IL18RAP* were associated with increased risk of NKTCL [8,9]. Li et al. provided the first evidence of a common SNP at *HLA-DPB1* contributing to NKTCL risk, with each copy of the risk allele raising the disease risk by 1.84 fold compared with the baseline wild-type genotype. This risk modulation may be mediated by alteration of antigen recognition and tumor clearance, due to variation at the binding pocket of the HLA-DPB1 protein (amino acid positions 84–87) [8]. In a larger follow-up study on 1417 NKTCL cases and 20,402 controls, Lin et al. subsequently identified two novel loci significantly associated with NKTCL development, independent of the risk conferred by *HLA-DPB1*, implicating inflammation and immune regulation through the IL18–IL18RAP axis and antigen presentation involving HLA-DRB1 [9]. The relevance of the immune microenvironment in NKTCL pathogenesis is further supported by the identification of recessive germline SNPs in *FAM160A1* reported in a pair of male siblings from a non-consanguineous Chinese family who were both diagnosed with NKTCL. FAM160A1 was expressed in CD68-positive histiocytes rather than the tumor cells, suggesting that variant FAM160A1 contributes to NKTCL pathogenesis by immune dysregulation [10].

Next generation sequencing has led to a greater clarity of the genomic landscape of NKTCL, revealing recurrent somatic mutations such as *TP53*, *JAK3*, *STAT3*, and *DDX3X* in NKTCL [11,101,102,103]. Additionally, frequent somatic *PD-L1* structural rearrangements have also been recently reported [104]. However, only mutational status of the *DDX3X*, *TP53*, and *KMT2D* genes were found to be prognostic in patients treated with CHOP-based regimens [11,12]. Molecular subtyping based on transcriptomics was able to group NKTCLs into three subtypes with association to survival: Tumor-suppressor/immune-modulator (TSIM), MYC-related (MB), and Histone epigenetic altered (HEA) groups. The NKTCL tumors in the HEA and MB subgroups were associated with the best and worst overall survival and progression-free survival, respectively. The associations were also made more pronounced when compared within the advanced Ann Arbor stage patients with NKTCL [13]. Aside from somatic mutations, a composite 7-single nucleotide polymorphism (7-SNP) germline signature was recently developed for predicting the survival of patients with NKTCL. The composite 7-SNP signature was suggested to be an additional risk indicator in helping clinical decision making [14].

## 6. Current Treatment Strategies for NKTCL and Potential Predictive Biomarkers

The treatment of disseminated NKTCL has entered a paradigm shift from anthracycline/CHOP-based regimens, to asparaginase-based regimens and to the most recent immunotherapy with PD-L1/PD1 immune checkpoint blockade [105]. Contemporary first-line therapeutic options for advanced NKTCL utilizes L-asparaginase as the backbone, including regimens such as SMILE [2,3], P-GEMOX [4], DDGP [5], and AspaMetDex [6]. However, patient outcomes remain poor with relapse rates approaching 70%. Upon relapse, salvage treatment using platinum-based or gemcitabine-based regimens suffer from limited efficacy [1]. As such, novel strategies incorporating immune checkpoint inhibitors and HDAC inhibitors have been explored in NKTCL. Blockade of the PD1/PDL1 axis in NKTCL has recently emerged as a promising treatment strategy. The combined objective response rate of three case series of patients with NKTCL being treated with pembrolizumab (PD-1 antibody) and avelumab (PD-L1 antibody) was as high as 54.3% (19/35) [106,107,108]. In an interim report, a phase II prospective study using sintilimab (PD-1 antibody) achieved an overall response rate of 68% in 28 relapsed or refractory NKTCL patients [109]. While these results are encouraging, predictive biomarkers are urgently required to help with patient stratification and treatment guidance.

In a recent study on the tumor immune microenvironment of NKTCL, Cho et al. analyzed the expression of 579 immune-related genes and characterized the immune cells using immunohistochemistry for FoxP3, PD-L1, and CD68. Four subgroups were identified, namely immune-tolerant (high T-reg cells and FOXP3 expression, early stage, best prognosis), immune evasion-A and immune evasion-B (high cytotoxic T-cells, high PD-L1, low T-reg), and immune-silenced (depleted immune response). The immune-silenced group represented patients with advanced disease and poor prognosis. Responses to pembrolizumab were observed for patients in the immune-tolerant group (1 out of 1) and immune-evasion group (3 out of 5) but none in the immune-silenced group (n = 5) [15]. Subsequently, Lee et al. identified *EGR1* upregulation in localized stage, low risk NKTCL. *EGR1* expression was highest in the immune-tolerant group and tended to decrease in the order of immune evasion-A, immune evasion-B, and immune-silenced subgroups, suggesting that *EGR1* contributes to early tumorigenesis by suppressing the immune response [110]. A retrospective multicenter clinicopathologic and genetic analysis of patients with relapsed or refractory NKTCL treated with pembrolizumab identified cryptic rearrangements of the PD-L1 gene disrupting the 3′-UTR to be a more reliable biomarker of the response to pembrolizumab than the immunohistochemical staining of membranous PD-L1 on NKTCL tumoral cells [16].

## 7. Developing Next Generation Biomarkers for NKTCL

Advances in next generation sequencing have led to the development of several novel molecular prognostic models and identification of potential predictive biomarkers of checkpoint immunotherapy in NKTCL. Liquid biopsy-based approaches may represent a non-invasive means of obtaining this information, bypassing the need for an invasive tissue biopsy [111]. In particular, plasma circulating tumor DNA (ctDNA) has recently come to the forefront as a dynamic marker reflecting disease burden, and represents a non-invasive means for disease assessment. In a recent study, Li et al. showed that the mutation spectrum of NKTCL could be adequately captured, and that serial monitoring could be useful for tracking residual disease. Furthermore, mutations in *KMT2D* and *ATM* were able to predict adverse outcomes [112].

MicroRNAs (miRNA) are small, noncoding RNAs that inhibit the translation of target genes and have been shown to play clinically important roles in NKTCL. For example, early studies showed that miR-21 and miR-155 were aberrantly overexpressed in NKTCL and activated oncogenic AKT signaling [113,114]. Low miR-146a expression was demonstrated to be an independent poor prognostic factor in NKTCL, while in NKTCL cell-lines, miR-146a overexpression inhibited NFκB activity, suppressed cell proliferation, induced apoptosis, and enhanced chemosensitivity, suggesting it functions as a tumor suppressor [115]. Reduced miR-15a expression, possibly mediated by EBV LMP1, was associated with poor prognosis [116]. In line with these findings, circulating microRNA profiles have also been investigated for their potential clinical utility. Circulating plasma miR-221 was associated with poor overall survival outcomes [117]. Plasma miR-155 was higher in non-responders to treatment compared with responders, and was directly correlated with serum expression of IL-6, IL-13, and TNF-α [118]. Increased serum levels of exosomal miR-4454, miR-21-5p, and miR-320e were also associated with poor overall survival [119]. Circulating EBV-encoded miRNAs miR-BART2-5p, miR-BART7-3p, miR-BART13-3p, and miR-BART1-5p discriminated NKTCL patients from healthy controls and significantly decreased after treatment. In addition, a high miR-BART2-5p level was associated with disease progression and poor prognosis [120].

## 8. Conclusions and Prospects

Moving forwards, high definition profiling technologies, including single cell sequencing and spatial transcriptomic assays, are expected to further unravel the complexities between tumor cells and the microenvironment of NKTCL, which may be especially relevant as immunotherapy evolves to the forefront of NKTCL treatment. The prospect of being able to dissect the molecular landscape of NKTCL at high resolution and the discovery of novel biomarkers are truly exciting as we enter a new era of precision medicine and immune oncology.

## Figures and Tables

**Table 1:** Summary of recent studies on molecular biomarkers in NKTCL.

Reference	Biomarker Type	Study Design	Main Findings
[8]	Diagnostic	GWAS	Germline SNPs at the loci of the *HLA-DPB1* locus associate with increased risk of NKTCL
[9]	Diagnostic	GWAS	Germline SNPs at two novel loci of *HLA-DRB1* and *IL18RAP* identified to confer risk of NKTCL
[10]	Diagnostic	Pedigree analysis and WES	Recessive germline SNPs in *FAM160A1* reported in male siblings who were both diagnosed with NKTCL
[11]	Prognostic	WES and targeted sequencing	Identified recurrent mutations in *DDX3X* and TP53, both conferring worse OS and PFS.
[12]	Prognostic	Targeted sequencing	Mutations in *KMT2D* and *TP53* associated with worse survival outcomes, and may enhance the prognostic value of the IPI model
[13]	Prognostic	Multi-omic profiling	Identified three molecular subtypes—Tumor-suppressor/immune-modulator (TSIM), MYC-related (MB) and Histone epigenetic altered (HEA) groups. HEA and MB subtypes were associated with the best and worst OS and PFS, respectively.
[14]	Prognostic	SNP genotype microarray	A 7-SNP-based classifier predicts the survival of patients with NKTCL, and improves existing risk stratification systems based on clinicopathological variables
[15]	Prognostic	RNA expression (NanoString) and IHC	Identified four immune subgroups—immune-tolerant (high T-reg cells and FOXP3 expression, early stage, best prognosis), immune evasion-A and immune evasion-B (high cytotoxic T-cells, high PD-L1, low T-reg), and immune-silenced (depleted immune response). The immune-silenced group represented patients with advanced disease and poor prognosis.
[16]	Predictive	WGS	Identified cryptic rearrangements of the *PD-L1* gene disrupting the 3′-UTR to be a potential predictive biomarker of response to pembrolizumab in relapsed or refractory NKTCL

Abbreviations: GWAS, Genome-wide association study; WES, whole exome sequencing; SNP, single-nucleotide polymorphism; OS, overall survival; PFS, progression-free survival; IHC, immunohistochemistry; WGS, whole genome sequencing.
